# Development and Validation of a 3-Plex RT-qPCR Assay for the Simultaneous Detection and Quantitation of the Three PML-RARa Fusion Transcripts in Acute Promyelocytic Leukemia

**DOI:** 10.1371/journal.pone.0122530

**Published:** 2015-03-27

**Authors:** Zhanguo Chen, Yongqing Tong, Yan Li, Qingping Gao, Qiongyu Wang, Chaohong Fu, Zunen Xia

**Affiliations:** 1 Department of Clinical Laboratory, Renmin Hospital of Wuhan University, Wuhan, Hubei, China; 2 Department of Hematology, Renmin Hospital of Wuhan University, Wuhan, Hubei, China; Saint Louis University School of Medicine, UNITED STATES

## Abstract

Rapid diagnosis of acute promyelocytic leukemia (APL) with promyelocytic leukemia-retinoic acid receptor alpha (PML-RARa) contributes to a highly effective therapy with all-trans retinoic acid (ATRA). Real-time quantitative reverse transcription-polymerase chain reaction (RT-qPCR) is a valuable tool to diagnose APL with PML-RARa. However, a single RT-qPCR analysis, which is laborious and costly, has to be performed in three reactions to determine whether one of the three PML-RARa transcripts is present and to quantify the involved transcript. This paper describes a novel TaqMan MGB probe-based 3-plex RT-qPCR assay in a single reaction to detect simultaneously the three PML-RARa transcripts. Specific primers and probe were designed, and the results were further normalized to the Abelson gene. The detection results for the serially diluted plasmid indicate that the analytical sensitivity was 10 copies per reaction for PML-RARa bcr1, bcr2, and bcr3. A relatively high sensitivity of 10^-4^ was achieved with this assay when analyzing the bcr1 transcripts obtained from the NB4 cell line. The reproducibility was satisfactory because the coefficients of variation of cycle threshold values were less than 3% for both inter- and intra-assays. After testing 319 newly diagnosed patients with leukemia (including 61 APL cases), the results of the 3-plex RT-qPCR assay completely agreed with the traditional methods used for the detection of PML-RARa. The quantitative results of the 3-plex RT-qPCR were highly correlated with the single RT-qPCR and showed similar assay sensitivity for 60 PML-RARa positive APL samples at diagnosis and 199 samples from 57 patients during follow-up. Interestingly, one PML-RARa bcr2 case at diagnosis with breakpoint at 1579, which was not detected by the single RT-q-PCR, was detected by the 3-plex RT-qPCR assay. The 3-plex RT-qPCR assay is a specific, sensitive, stable, and cost-effective method that can be used for the rapid diagnosis and treatment monitoring of APL with PML-RARa.

## Introduction

The presence of t(15;17)(q22;q21) with the promyelocytic leukemia-retinoic acid receptor alpha (PML-RARa) fusion gene is regarded as the hallmark of acute promyelocytic leukemia (APL), characterized by a translocation involving the fusion of the promyelocytic leukemia (PML) gene at 15q22 with retinoic acid receptor alpha (RARa) gene at 17q21 [1–3]. Coagulopathy manifestations are the major cause of death in APL patients if appropriate treatment regimen is not promptly administered [4–6]. Rapid detection of the PML-RARa fusion gene provides the molecular basis for a highly effective therapy with all-trans retinoic acid (ATRA) and arsenic trioxide [4,7–9]. The formation of the three PML-RARa transcript subtypes depends on the location of breakpoints within the PML gene (intron 6, exon 6, and intron 3) and within the RARa gene (intron 2); these subtypes referred to as long (L or bcr1), variant (V or bcr2), and short (S or bcr3) account for 55%, 5%, and 40% of the cases, respectively [10].

At present, the molecular diagnosis of PML-RARa positive APL cases is mainly based on the result of karyotyping, FISH, and reverse transcription-polymerase chain reaction (RT-PCR). Among these techniques, RT-PCR seems to be the only approach suitable for the detection of PML-RARa transcripts and minimal residual disease (MRD) evaluation [10–12]. However, conventional RT-PCR has limitations, including possible failure in identifying poor-quality samples that can potentially cause false-negative results and inadequate capacity to distinguish between increasing and decreasing levels of PML-RARa transcripts. Real-time quantitative reverse transcription-polymerase chain reaction (RT-qPCR) not only provides information on the relationship between different levels of disease at early phases of therapy, but also monitors MRD to predict relapse, guiding early intervention to prevent disease progression [13–16]. To date, multiple primers and probes, which can detect various PML-RARa fusion transcripts at one time, have been designed for the RT-qPCR [17,18]. According to the Europe Against Cancer (EAC), single RT-qPCR protocol is the most representative method for the quantification of PML-RARa transcripts [18]. This protocol has to be performed in three reactions to determine whether one of the three PML-RARa transcripts is present and to quantify the involved transcript. However, single RT-qPCR may be laborious and costly because more primers, probes, and PCR reactions are necessary for the identification of PML-RARa transcripts. Moreover, the EAC forward primer for PML-RARa bcr2 (ENF906 PML) [18] is located from nucleotide 1642 to nucleotide 1660 on PML exon 6 according to accession number M73778. As a consequence, this test can lead to false-negative results for some rare variants of PML-RARa bcr2 with breakpoints located 5′ to nucleotide 1642.

Basing on the critical breakpoint of PML-RARa bcr2, we established a novel TaqMan MGB probe-based 3-plex RT-qPCR assay to simultaneously detect the three PML-RARa transcripts found in APL patients. After evaluating the diluted positive control and clinical samples, the assay exhibited favorable sensitivity, specificity, and reproducibility. Quantitative results of the 3-plex RT-qPCR were highly correlated with the results from single RT-qPCR and showed similar assay sensitivity for most of the PML-RARa positive APL samples at diagnosis and all samples during follow-up, except for one PML-RARa bcr2 case at diagnosis with breakpoint at 1579. This assay is an easy, efficient, reliable, and cost-effective method that can be used for fast molecular diagnostics of suspected APL and MRD monitoring of the patients with APL.

## Patients, Materials and Methods

### Patients, Biological Materials, and Controls

The present study included 319 leukemia patients who were referred to the Department of Clinical Laboratory at Renmin Hospital of Wuhan University between January 2010 and August 2014 for clinical diagnostic purposes. Diagnosis of patients was established according to French American British (FAB) and 2008 World Health Organization criteria [19,20]. Bone marrow (BM) samples were collected during initial diagnosis. The remaining diagnostic materials were obtained from the patients without any additional sampling and after all the available techniques for the diagnosis of their pathology had been performed. Once patients were identified as APL, BM samples during follow-up were requested for RT-qPCR analysis after induction and 3 cycles of consolidation therapy according to the treatment protocol [15,21] and International APL Guideline [4]. MRD status was assessed after each treatment course. Each sample was sent to an independent laboratory for the assessment of the status of fusion genes as part of the validation process by a conventional nested RT-PCR [22]. PML-RARa transcripts were simultaneously amplified using EAC single RT-qPCR assay and a novel 3-plex RT-qPCR assay run on ABI7500 Real Time PCR System (Applied Biosystems, Foster City, USA) according to EAC protocol [18] and on ViiA7 Real Time PCR System (Applied Biosystems, Foster City, USA), respectively. Sequencing was performed on the PML-RARa bcr2 positive samples to determine the exact location of the involved breakpoint. The primary characteristics of these patients are listed in Table 1. Eighty-nine healthy donors were recruited as controls, and peripheral blood (PB) samples were collected from them. Approval was obtained from the ethics committee of Renmin Hospital of Wuhan University. Written informed consent was obtained from all patients and controls in accordance with the Declaration of Helsinki.

**Table 1:** Primary characteristics of 319 patients with leukemia.

Characteristic	No.(%)
Patients	319
Sex	
Male	189 (59.1)
Female	130 (40.9)
Age (years)	
>60	105(32.8)
18 to 60	142(44.6)
<18	72 (22.6)
WHO classification	
Acute myeloid leukemia and related neoplasms (AML)	201 (63.0)
AML with t(8;21)(q22;q22);RUNX1-RUNX1T1	39 (12.2)
AML with inv(16)(p13.1q22) or t(16;16)(p13.1;q22); CBFB-MYH11	32 (10.0)
APL with t(15;17)(q22;q12);PML-RARA	61 (19.1)
AML with t(9;11)(p22;q23);MLLT3-MLL	4 (1.3)
AML with t(6;9)(p23;q34);DEK-NUP214	1 (0.3)
AML with myelodysplasia-related changes	19 (6.0)
AML, not otherwise specified	41 (12.9)
AML with therapy-related myeloid neoplasms	4 (1.3)
Chronic myelogenous leukemia (CML), with positive BCR-ABL1	51 (16.0)
B lymphoblastic leukemia/lymphoma (B-ALL)	54 (16.9)
B-ALL with t(9;22) (q34;q11.2);BCR-ABL1	10 (3.1)
B-ALL with t(v;11q23);MLLrearranged	12 (3.8)
B-ALL with t(12;21)(p13;q22);TEL-AML1	14 (4.4)
B-ALL with t(1;19)(q23;p13.3);TCF3-PBX1	16 (5.0)
Other B-ALL, NOS	2 (0.6)
T lymphoblastic leukemia/lymphoma (T-ALL)	13 (4.1)

The NB4 cell line with t(15;17) and PML-RARa bcr1 transcripts was used as a positive control [23]. The positive controls for the PML-RARa bcr3 and bcr2 transcripts were derived from positive patient samples carrying the t(15;17) short and variant forms, respectively. The K562 cell line was used as a negative control. All cells were cultured in RPMI 1640 medium supplemented with 10% fetal bovine serum (Invitrogen, Grand Island, NY, USA) in a 37°C incubator with 5% CO_2_.

### RNA Isolation and cDNA Synthesis

From heparinized BM or PB samples, mononuclear cells (MNC) were obtained by Ficoll-Hypaque density centrifugation, whereas the cell lines were collected via centrifugation. Total RNA from MNC and cell line samples were extracted with TRIzol reagent according to the manufacturer’s procedure (Life Technologies, Carlsbad, USA). Total RNA was extracted, isolated, and diluted in 30 μL of DEPC-treated water, and the concentration of total RNA was then measured using a NanoDrop 2000 (NanoDrop Technologies, Wilmington, DE, USA). Total RNA (≤2 μg) was reverse transcribed to cDNA in a total reaction volume of 20 μL using 5 μmol/L Oligo(dT)_18_ primers, 1× reaction buffer, 1 U/μL RNase inhibitor, 500 μmol/L each dNTP, and 10 U/μL M-MuLV reverse transcriptase following the manufacturer’s protocol (RevertAid First Strand cDNA Synthesis Kit, Thermo Scientific). The reverse transcription reaction was performed at 42°C for 60 min and then terminated by heating at 72°C for 5 min. Aliquots were stored at −80°C prior to analysis.

### Primer and Probe Design

**Fig 1:**
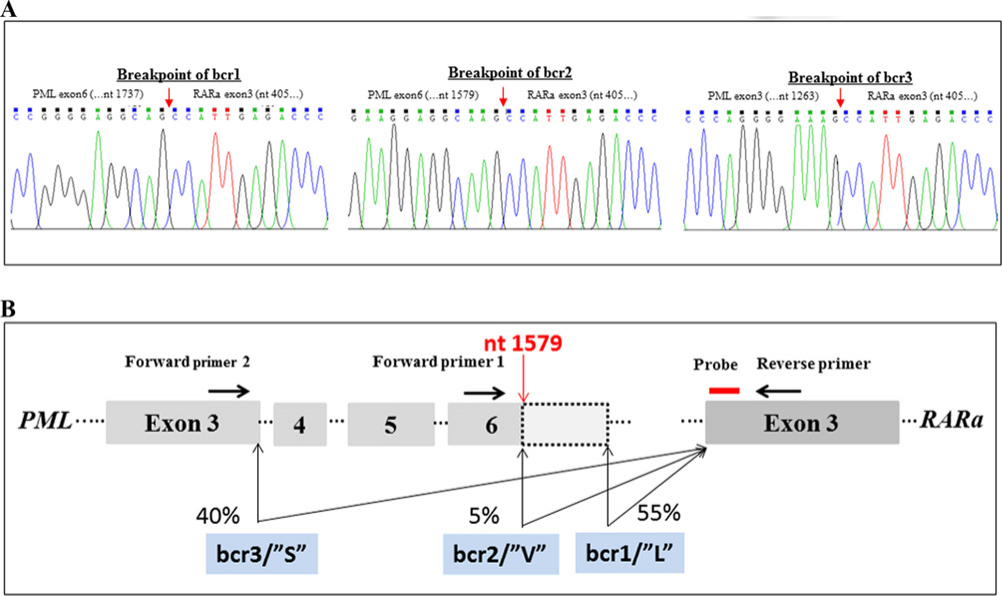
Primers and probe selection based on the breakpoint of PML-RARa. (A) The sequence of breakpoint of PML-RARa bcr1, bcr2 and bcr3. (B) Location of primers and probes for detection of all PML-RARa with respect to PML breakpoint. Forward primer 1 (bcr1 and bcr2) and Forward primer 2 (bcr3) were used in combination with the common Reverse primer and probe to detect three PML-RARa transcripts. Details of primers and probe sequences are provided in Table 2. The short red arrow on Fig. 1A indicates breakpoints of PML-RARa bcr1, bcr2 and bcr3, respectively. The long red arrow on Fig. 1B indicates breakpoint of PML-RARa bcr2. Abbreviations: S, short isoform; L, long isoform; V, variable breakpoint. The PML gene and RARA gene are positioned according to accession numbers M73778 (PML) and X06538 (RARA).

One of the main obstacles in developing a 3-plex RT-qPCR assay for the simultaneous detection of PML-RARa bcr1, bcr2, and bcr3 was primer placement for the variant PML-RARa bcr2. A forward primer or a single probe placed in the variable region of PML exon 6 may lead to false-negative results. We used the same primer to detect both transcripts simultaneously because PML-RARa bcr1 and bcr2 contain at least an upstream portion of PML exon 6. Thus, primers and probe were designed for the three PML-RARa transcripts such that all previously reported breakpoints could be detected [24–26]. We sequenced the PML-RARa bcr2 positive samples, one of which had an aberrant breakpoint at nucleotide 1579 of PML exon 6 (Fig. 1A). Breakpoints of PML-RARa bcr1, bcr2, and bcr3 are shown in Fig. 1A. Three primers and one probe set for the 3-plex RT-qPCR assay were designed: one forward primer for PML-RARa bcr3, one forward primer for PML-RARa bcr1 and bcr2, and the same reverse primer and probe for the simultaneous detection of the three PML-RARa transcripts (Fig. 1B). Primers and TaqMan MGB probes were designed using Primer Express software 3.0 (Applied Biosystems) and Primer blast (http://www.ncbi.nlm.nih.gov/tools/primer-blast/) according to the manufacturer’s guidelines. Sequences of primers and probes for RT-qPCR and primers for RT-PCR are listed on Table 2. We selected Abelson (ABL) as the reference gene to assess the quality of RNA samples, and the levels of PML-RARa in the patient samples were further normalized to the ABL gene.

**Table 2:** Primers and probes for 3-plex RT-qPCR of PML-RARa and ABL.

Primer/probe^a^	Sequence (5′-3′)	GenBank Accession No.	Position
RT-PCR for PML-RARa			
PML primer 1	AGAGGATGAAGTGCTACGCCT	M73778	1081–1101
RARA primer 2	TTCCGGGTCACCTTGTTGAT	X06538	620–639
RT-PCR for ABL			
ABL primer 3	GGGCTGTCCTCGTCCTCCA	X16416	193–211
ABL primer 4	CACCCTCCCTTCGTATCTCA	X16416	641–660
3-plex RT-qPCR for PML-RARa			
PML primer 5	CCCCAGGAAGGTCATCAAGA	M73778	1532–1551
PML primer 6	GACCTCAGCTCTTGCATCACC	M73778	1233–1253
RARA primer 7	GGCTTGTAGATGCGGGGTAG	X06538	486–467
RARA probe (r)	FAM-TAGTGCCCAGCCCTCC-MGB	X06538	438–453
3-plex RT-qPCR for ABL			
ABL primer 8	ACCCAGTGAAAATGACCCCA	X16416	318–327
ABL primer 9	GATGTAGTTGCTTGGGACCCA	X16416	475–495
ABL probe (r)	VIC-TGGGCTTCACACCATTC-MGB	X16416	439–455

^a^Probe (r) = Reverse TaqMan MGB probe; PML primer 1 for bcr1, bcr2 and bcr3; PML primer 5 for bcr1 and bcr2; PML primer 6 for bcr3

### Preparation of Standards

Two pairs of universal primers (Table 2) were used to amplify the three PML-RARa transcripts and the ABL control gene from the positive samples. Amplified PCR products were then cloned into pGEM-T Easy vectors (Promega, Madison, USA). The plasmid DNA of selected clones was purified using AxyPrep Plasmid Miniprep Kit (AXYGEN, Union City, USA) and sequenced by Big Dye Terminator Cycle Sequencing Kit (Applied Biosystems, Carlsbad, USA) using ABI Prism 3130 sequence detection system to confirm the insert. The confirmed plasmids were measured by a NanoDrop spectrophotometer, and the molecular concentrations were calculated according to the molecular weights of the vector and the insert. Ten-fold series dilution of plasmid (10^6^, 10^5^, 10^4^, 10^3^, 10^2^, and 10 copies in 2 μL) was used for the standard curves. PCR efficiencies were calculated from the slope of the curves produced from the diluted samples (PCR efficiency=(10−1slope−1)×100%).

### Optimization of the 3-plex RT-qPCR

PML-RARa and ABL were amplified in separate reactions at the same time. We selected various PCR programs, reaction volumes, and different concentrations of primers, probe, MgCl_2_, dNTPs, and TaKaRa Taq HS to optimize the RT-qPCR after extensive testing on the NB4 cell line, patient samples, and serial-diluted plasmids. The optimized amplification reactions (25 μL) for PML-RARa contained 1× buffer, final concentrations of 4.5 mmol/L MgCl_2_, 0.3 mmol/L dNTPs, 200 nmol/L of each primer, 100 nmol/L FAM-labeled MGB probe for PML-RARa, 1.25 U TaKaRa Taq HS, and 2 μL of cDNA or plasmid templates. The amplification reactions (25 μL) of ABL were the same as those of PML-RARa, except for 200 nmol/L of each primer and 100 nmol/L VIC-labeled probe. Reactions were performed on the ViiA7 Real Time PCR System (Applied Biosystems, Foster City, USA). The closed tubes were incubated at 50°C for 2 min and pre-denatured at 95°C for 3 min, followed by 45 cycles of denaturing at 95°C for 5 s and annealing and elongation at 58°C for 45 s. Real-time fluorescent data of FAM and VIC were collected and analyzed with ViiA 7 Software v1.2.3.

### Amplification of PML-RARa Transcripts in APL

PML-RARa and ABL were separately amplified from the same cDNA using the optimized 3-plex RT-qPCR assays. Quantification was performed using the standard curves, prepared as 10-fold serial dilutions in deionized water from 10^6^ to 10 copies in 2 μL. The level of PML-RARa transcripts in patients was further normalized to the reference gene ABL as the normalized copy number (NCN). The 3-plex RT-qPCR assay was tested on 319 BM samples from leukemia patients at initial diagnosis (including 61 patients with APL), 89 PB samples from healthy controls and 199 BM samples from 57 patients with APL during follow-up. All experiments were performed in triplicate, and a valid result required a threshold cycle (Ct) value of ABL within a range of 20 to 30, with at least 2,000 copies of the ABL gene in the sample. In particular, no-amplification controls that contained known negative controls and no-template controls (NTC) that contained water instead of human cDNA were used as two types of negative controls to detect cross-contamination of PCR products. Single RT-qPCR analysis of PML-RARa transcripts for BM samples of patients with APL at diagnosis and during follow-up was simultaneously performed according to the standardized EAC protocols [18]. The correlation between 3-plex RT-qPCR and single RT-qPCR assays was calculated.

### MRD and Sensitivity Calculation

Threshold fluorescence was set at 110000 and 80000 for PML-RARa and ABL, respectively. For PML-RARa, we defined Ct = 40 as our detection limit, and Ct values above this limit were defined as negative. The mean Ct values and the mean value of the copy number (CN) for PML-RARa and ABL were used. For relative MRD calculation, we employed the NCN method and NCN was defined as the CN of the PML-RARa per one copy of ABL control gene (i.e., PML-RARa copies/ABL copies). MRD value was presented as absolute value using the equation:

$$MRD\;value=\frac{{\left(PML-RARa\;copies/ABL\;copies\right)}_{Follow-up}}{{\left(PML-RARa\;copies/ABL\;copies\right)}_{Diagnosis}}\times 100\%$$ (1)

The assay sensitivity at diagnosis was calculated as follows:

$$Sensitivity_{Diagnosis}=-log_{10}(NCN)-log_{10}(ABLcopies)$$ (2)

The assay sensitivity during follow-up was calculated using the equation:

$$Sensitivity_{Follow-up}=log10\left[\frac{{\left(ABLcopies\right)}_{Diagnosis}}{{\left(ABLcopies\right)}_{Follow-up}\text{×}{\left(PML-RARacopies\right)}_{Diagnosis}}\right]$$ (3)

according to EAC formula for calculation of MRD value and theoretical sensitivity [18,27].

### Definitions and Statistical Analysis

Hematologic complete remission (CR) was defined according to standard criteria [28]. Molecular CR was defined when PML-RARa fusion transcripts were not detectable by RT-qPCR in a BM sample that afforded a sensitivity of at least 1 in 10^3^. Fisher’s exact test was used to compare two groups of categorical clinical variables. For the continuous variables, nonparametric Mann—Whitney U test was employed. The correlation of PML-RARa/ABL NCN results between 3-plex RT-qPCR and single RT-qPCR was calculated by using the Spearman’s rho coefficient. All statistical analyses were performed with SPSS 19.0 for Windows (SPSS, Chicago, USA). *P* < 0.05 was regarded to be statistically significant.

## Results

### Optimization of the 3-plex RT-qPCR and Selection of Standard Curve

**Fig 2:**
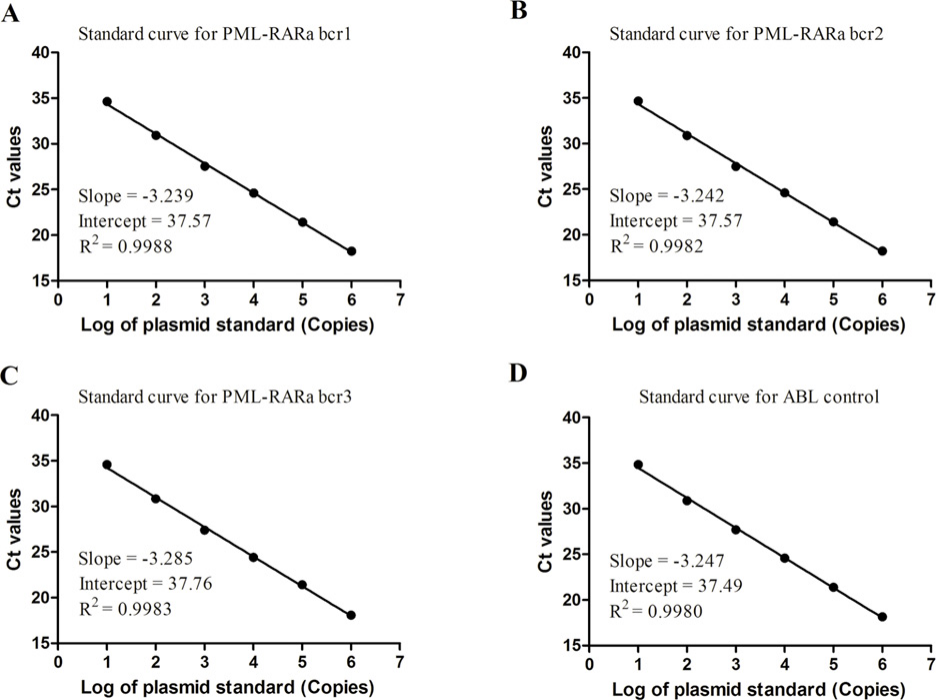
Standard curve for PML-RARa bcr1, bcr2, bcr3 and ABL control gene. (A) Standard curve for PML-RARa bcr1. (B) Standard curve for PML-RARa bcr2. (C) Standard curve for PML-RARa bcr3. (D) Standard curve for ABL control. The range on all curves is from 10^6^ to 10 copies of corresponding plasmids. The efficiencies of PML-RARa bcr1, bcr2 and bcr3 were found to be similar to that of ABL, with slope values much closer to -3.32 (the slope associated with a PCR reaction with maximal efficiency).

The optimized conditions for 3-plex RT-qPCR were described in the methods in detail. The amplification plots using serially diluted plasmid standards for PML-RARa bcr1, bcr2, bcr3, and ABL demonstrated a high degree of similarity (data not shown). The four standard curves yielded a very consistent Ct for each point with correlation coefficients more than 0.99 (Fig. 2A-2D). This result indicates that the linearity ranged over six orders of magnitude from 10^6^ to 10 copies/2 μL. The practical slopes of the three standard curves and ABL standard curve were close to -3.32 for PCR reaction with maximum efficiency (Fig. 2). This finding demonstrates that the four assays displayed similar kinetics and optimal PCR amplification. The efficiencies of RT-qPCR for PML-RARa bcr1, bcr2, bcr3, and ABL were 103.6%, 103.4%, 101.6%, and 103.2%, respectively, which were confirmed by serial diluted clinical samples with high concentration of PML-RARa (data not shown). Moreover, the RT-qPCR for PML-RARa bcr1, bcr2, bcr3, and ABL provided reliable quantification within the diagnostic range (from 10^6^ to 10 copies/2 μL) with high correlation coefficient between the Ct value and concentration. Therefore, we selected one of the PML-RARa standard curves (i.e., PML-RARa bcr1) and the ABL standard curve to determine the precise amount of target present in the test samples.

### Specificity and Analytical Sensitivity

The specificity of 3-plex RT-qPCR assay was determined by testing 319 BM samples with t(15;17) or other translocations. The conventional nested RT-PCR and/or karyotype method detected 240 rearrangements in the 319 patients with leukemia at diagnosis: 61 PML-RARa, 39 RUNX1-RUNX1T1, 32 CBFB-MYH11, 4 MLLT3-MLL, 1 DEK-NUP214, 61 BCR-ABL1, 12 MLL rearranged, 14 TEL-AML1, and 16 TCF3-PBX1. The results of the 3-plex RT-qPCR from the 319 diagnostic leukemia patient samples were consistent with those of the traditional methods used for detection of the three PML-RARa transcripts associated with t(15;17) (Table 3).

**Table 3:** Comparative results for 3-plex RT-qPCR and other methods from 319 patients with leukemia for transcripts of PML-RARa.

Translocation/rearrangement	Patients	Karyotype	Conventional nested RT-PCR	3-plex RT-qPCR
t(15;17)(q22;q12)/PML-RARA	61	61	61^a^	61
t(8;21)(q22;q22)/RUNX1-RUNX1T1	39	39	39	0
inv(16)(p13.1q22) or t(16;16)(p13.1;q22)/ CBFB-MYH11	32	32	32	0
t(9;11)(p22;q23)/MLLT3-MLL	4	4	4	0
t(6;9)(p23;q34)/DEK-NUP214	1	1	1	0
t(9;22)(p34;q11.2)/ BCR-ABL1	61	61	61	0
t(v;11q23)/MLL rearranged	12	12	12	0
t(12;21)(p13;q22)/TEL-AML1	14	14	14	0
t(1;19)(q23;p13.3)/TCF3-PBX1	16	16	16	0
No translocation/rearrangement	79	79	0	0
Total	319	319	240	61

^a^There were 33(54.1%) cases of PML-RARa bcr1, 3(4.9%) cases of PML-RARa bcr2 and 25 (41.0%) cases of PML-RARa bcr3.

**Fig 3:**
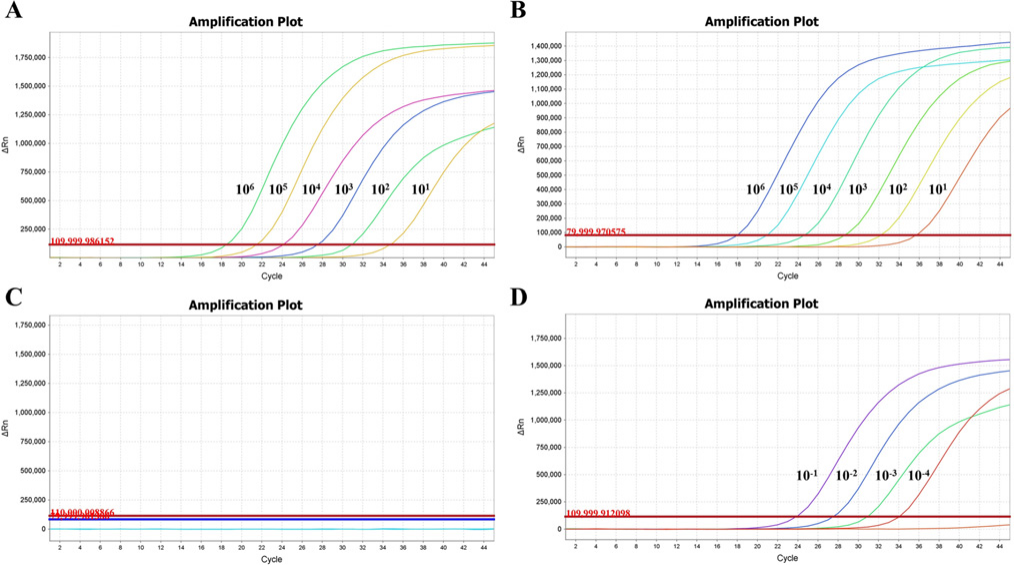
The analytical sensitivity of the PML-RARa 3-plex RT-qPCR assay. (A) Amplification plot for PML-RARa of serial plasmid dilutions with analytical sensitivity of 10 copies per reaction. (B) Amplification plot for ABL of serial plasmid dilutions with analytical sensitivity of 10 copies per reaction. (C) Amplification plot for no template control (NTC). (D) Amplification plot for 1μg RNA from PML-RARa positive NB4 cell line diluted in control RNA sample with analytical sensitivity of 10^-4^ (1 in 10^4^) dilution. Dilutions of PML-RARa and ABL ranged from 10^6^ to 10^0^ copies, and dilutions of NB4 cell RNA ranged from 10^-1^ to 10^-5^.

Of the 319 BM samples from patients and 89 PB samples from healthy controls, the 3-plex RT-qPCR exclusively detected the PML-RARa transcript in 61 BM samples from APL patients at diagnosis, demonstrating 100% specificity. This result indicates that the 3-plex RT-qPCR assay can detect the three PML-RARa transcripts without cross-reaction with the other fusion genes. The analytical sensitivity of the 3-plex RT-qPCR assay was determined by analyzing 10 replicates of serial 10-fold plasmid dilutions (10^6^ to 1 copies) and serial dilutions of RNA from the NB4 cell line. The plasmid dilution of 10 copies/reaction was consistently detected by the assay, showing an analytical sensitivity of 10 copies for the PML-RARa plasmid (Fig. 3A) and 10 copies for the ABL plasmid (Fig. 3B). None of the NTC showed positive amplification plot (Fig. 3C). When the analytical sensitivity was further analyzed using serial 10-fold dilutions of 1 μg RNA from the NB4 cell line (10^-1^ to 10^-5^) diluted in the K562 cell line to maintain consistent quantities of background RNA throughout the dilution series, the assay achieved a sensitivity of 10^-4^ (Fig. 3D). Below this threshold, results were not reproducibly stable.

### Reproducibility of the Assay

**Fig 4:**
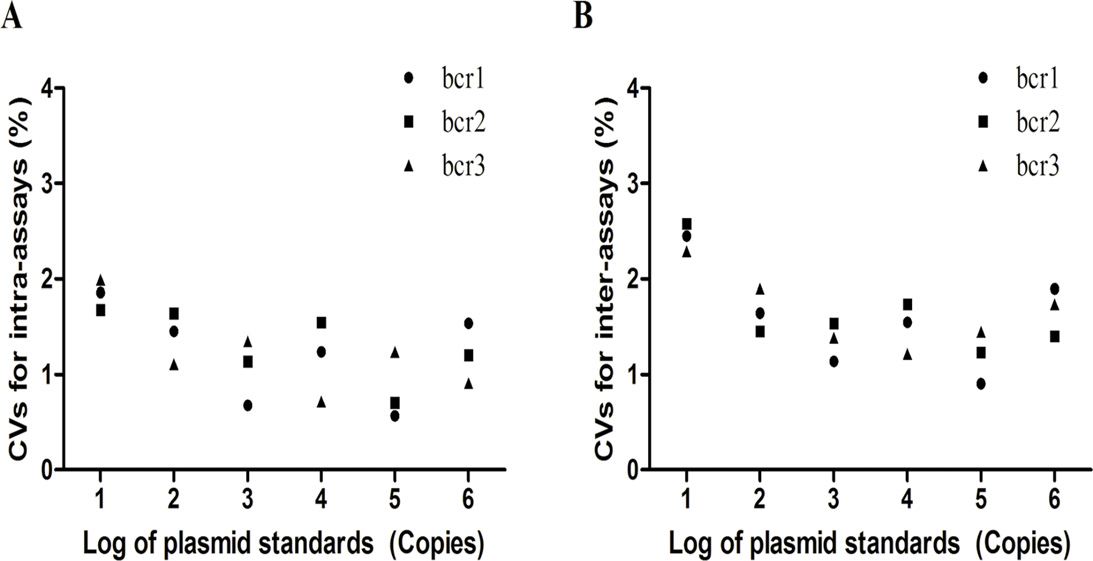
Intra- and inter-assay reproducibility of PML-RARa bcr1, bcr2 and bcr3 for 3-plex RT-qPCR assay. (A) The intra-assay reproducibility for bcr1, bcr2 and bcr3. (B) The inter-assays reproducibility for bcr1, bcr2 and bcr3. The inter- and intra-assay coefficients of variation (CVs) for serial dilution of plasmid standards of PML-RARa bcr1, bcr2, and bcr3 (from 10^6^ to 10 copies/2 μL). All inter-assay and intra-assay CVs were less than 3%.

We calculated the inter- and intra-assay coefficients of variation (CVs) for serially diluted plasmid standards of PML-RARa bcr1, bcr2, and bcr3 (from 10^6^ to 10 copies/2 μL) to evaluate the reproducibility of the assay. The intra-assay reproducibility of PML-RARa bcr1, bcr2, and bcr3, calculated from the Ct values of 10 replicates from a single run, demonstrated CVs ranging from 0.567% to 1.857% for bcr1, from 0.706% to 1.678% for bcr2, and from 0.717% to 1.990% for bcr3 (Fig. 4A). The inter-assay reproducibility of PML-RARa bcr1, bcr2, and bcr3, which was determined by comparing the Ct values from 10 different runs, had CVs ranging from 0.906% to 2.447% for bcr1, from 1.234% to 2.578% for bcr2, and from 1.217% to 2.290% for bcr3 (Fig. 4B). All inter- and intra-assay CVs were less than 3%, suggesting that this 3-plex RT-qPCR method is highly reproducible.

### Validation of the 3-plex RT-qPCR in Patients with Leukemia at Diagnosis

**Fig 5:**
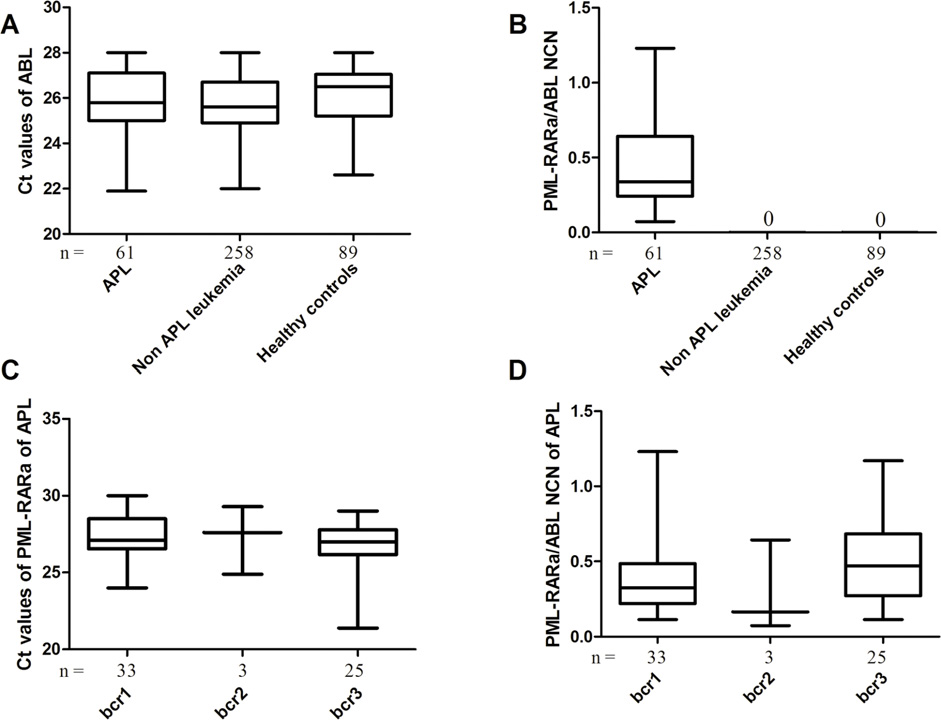
The results of 3-plex RT-qPCR in patients with leukemia at diagnosis. (A) Ct values of ABL for patients with leukemia at diagnosis and healthy controls. (B) PML-RARa/ABL NCN for patients with leukemia at diagnosis and healthy controls. (C) Ct values for APL patients with PML-RARa bcr1, bcr2 and bcr3. (D) PML-RARa/ABL NCN of APL patients with PML-RARa bcr1, bcr2 and bcr3. There was no significant difference among bcr1, bcr2 and bcr3 patients in the Ct values of PML-RARa (*P* > 0.05) and PML-RARa/ABL NCN regardless of age, gender, white blood cell count (WBC), hemoglobin, platelet count and percentage of promyelocytes (*P* > 0.05).

**Fig 6:**
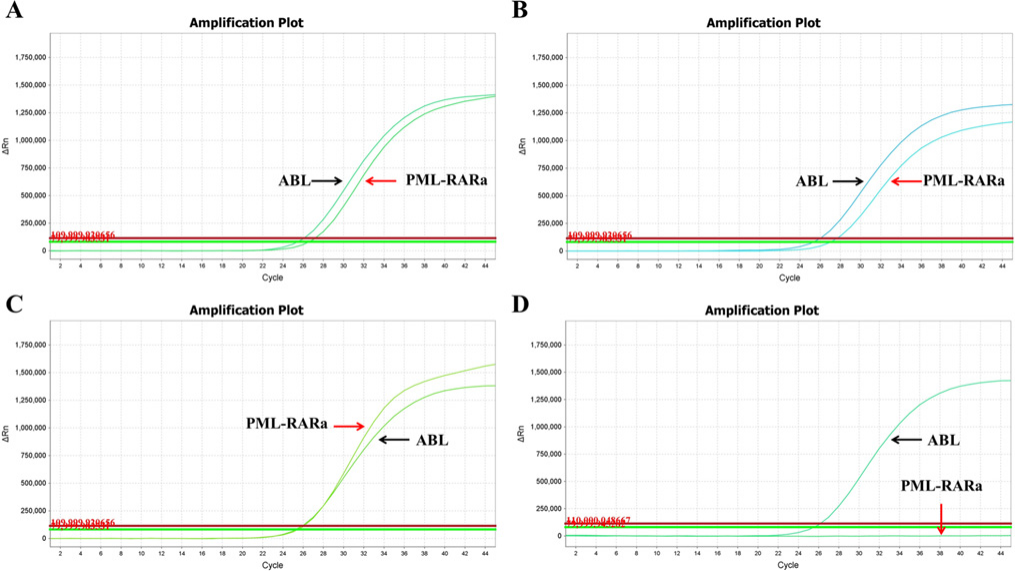
The representative amplification plots of PML-RARa bcr1, bcr2, bcr3 and control by 3-plex RT-qPCR. (A) Amplification plot for PML-RARa bcr1 positive samples. (B) Amplification plot for PML-RARa bcr2 positive samples. (C) Amplification plot for PML-RARa bcr3 positive samples. (D) Amplification plot for PML-RARa negative samples. The red arrow indicates the amplification curve of PML-RARa, whereas black arrow indicates the amplification curve of ABL control.

All the samples (319 patients with leukemia at diagnosis and 89 controls) showed amplification of the ABL control gene with Ct values ranging from 21 to 28, indicating a good quality of mRNA samples (Fig. 5A). The 3-plex RT-qPCR assay efficiently detected all the 61 PML-RARa positive samples (Fig. 5B), specifically, 33 with bcr1, 3 with bcr2, and 25 with bcr3 (Fig. 5C). Sequence analysis of the three PML-RARa bcr2 positive samples was performed to determine the breakpoint involved. Two PML-RARa bcr2 cases contained breakpoints at nucleotide 1685 (data not shown) and one case contained a breakpoint at nucleotide 1579 (Fig. 1A). No amplification was observed in non-APL patients and controls (Fig. 5B), and positive amplifications were only observed in APL patients with PML-RARa (Fig. 5B). Significant difference was not observed among bcr1, bcr2, and bcr3 patients in the Ct values of PML-RARa (Fig. 5C) and PML-RARa/ABL NCN (Fig. 5D) regardless of age, gender, white blood cell count, hemoglobin, platelet count, and percentage of promyelocytes (*P* > 0.05). We calculated the assay sensitivities at diagnosis for APL according to Equation (2). When the levels of ABL expression and PML-RARa/ABL NCN were considered, the median level of PML-RARa expression observed in 61 BM samples at diagnosis equated with an assay sensitivity of 10^−4.15^ (range, 10^−3.27^ to 10^−5.89^). The representative amplification plots of PML-RARa bcr1, bcr2, bcr3, and negative control by 3-plex RT-qPCR are presented in Fig. 6. The overall amplification plots from both PML-RARa and ABL demonstrated a high degree of similarity (Fig. 6A-6C), whereas the negative control generated an amplification plot for ABL (Fig. 6D), but not for PML-RARa (under the threshold). Although the quantification of PML-RARa transcript is not isoform-specific, complementary use of gel electrophoresis from 3-plex RT-qPCR can be done to further classify the subtypes of PML-RARa fusion transcripts (S1 Fig.).

### Comparison of 3-plex RT-qPCR Assay with Single RT-qPCR Assay at Diagnosis

**Fig 7:**
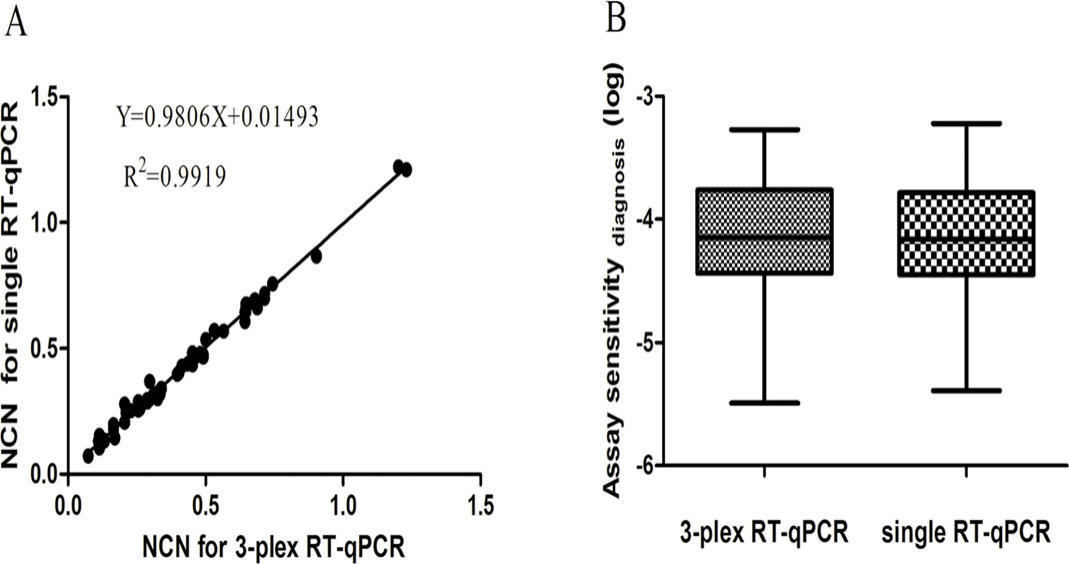
Comparison of 3-plex RT-qPCR assay with single RT-qPCR assay for detection of PML-RARa positive samples from 60 APL patients. (A) Correlation of PML-RARa/ABL NCN between 3-plex RT-qPCR assay and single RT-qPCR assay. (B) Sensitivity of detection for 60 APL samples by 3-plex RT-qPCR assay and single RT-qPCR assay.

To evaluate whether a difference in PML-RARa/ABL NCN exists between the 3-plex RT-qPCR and the single RT-qPCR, we analyzed 61 PML-RARa positive samples using both methods. The 3-plex RT-qPCR assay detected all the 61 PML-RARa positive samples, whereas the single RT-qPCR detected 60 PML-RARa positive samples except for one PML-RARa bcr2 case with breakpoint at 1579, suggesting the better capability of 3-plex RT-qPCR to detect the rare variants of PML-RARa bcr2. The results of both methods were highly correlated for the 60 PML-RARa positive samples (Spearman’s rho coefficient = 0.992, *P* < 0.01) (Fig. 7A). The slope of the regression curve did not differ significantly from 1, implying that both methods provided similar results. No significant difference was observed between 3-plex RT-qPCR and single RT-qPCR for the PML-RARa NCN values (*P* > 0.05). Meanwhile, assay sensitivity at diagnosis was calculated according to Equation (1). The 3-plex RT-qPCR assay achieved the median sensitivity of 10^−4.15^ (range, 10^−3.27^ to 10^−5.23^), whereas the single RT-qPCR assay achieved the median sensitivity of 10^−4.16^ (range, 10^−3.22^ to 10^−5.39^) (Fig. 7B). No significant difference was observed between both methods in assay sensitivity (*P* > 0.05).

### Application of 3-plex RT-qPCR Assay for MRD Monitoring

**Fig 8:**
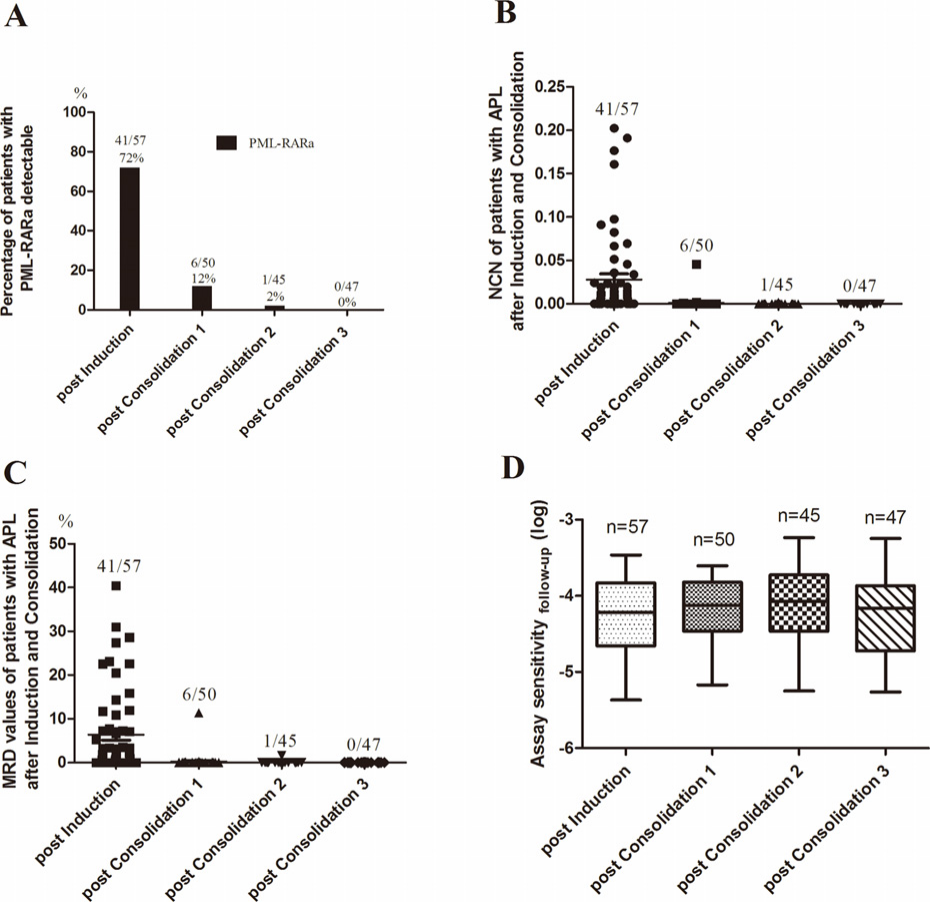
3-plex RT-qPCR for MRD detection in patients with APL treated with ATRA-based therapy after induction and 3 cycles of consolidation. (A) Percentage of APL patients with PML-RARa detectable after induction and consolidation. (B) PML-RARa/ABL NCN of patients with APL after induction and consolidation. (C) MRD assessment of patients with APL after induction and consolidation. (D) Assay sensitivity of APL samples after induction and consolidation. Post consolidation 1, after the 1st cycle of consolidation; Post consolidation 2, after the 2nd cycle of consolidation; Post consolidation 3, after the 3rd cycle of consolidation.

Of the 61 patients with APL initially included in the study, three patients died of cerebral hemorrhage and one patient died of pulmonary infection during induction. To assess whether the 3-plex RT-qPCR assay can be used for MRD monitoring, we detected the 199 consecutive available samples from 57 patients with APL after induction and 3 cycles of consolidation, including 57 BM samples after induction, 50 BM samples after the 1st cycle of consolidation, 45 BM samples after the 2nd cycle of consolidation and 47 BM samples after the 3rd cycle of consolidation. Though all patients achieved hematologic complete remission after induction, the majority of patients (72%) tested positive on 3-plex RT-qPCR (Figs. 8A and 8B). The minority of patients (12%) tested positive on 3-plex RT-qPCR after the 1st cycle of consolidation, and the majority of patients (98%) were in molecular CR after the 2nd cycle of consolidation except for one patient who achieved molecular CR after the 3rd cycle of consolidation (Fig. 8A and 8B). MRD values of patients during follow-up were assessed by Equation (1). Thirteen of 57 patients were above 10% level of MRD at timepoint of post induction (Fig. 8C). There were 6 of 50 patients with PML-RARa detectable at timepoint of post consolidation 1 (Fig. 8A and 8B), five of which was below 1% level of MRD (Fig. 8C). Most patients at timepoint of post consolidation 2 (98%) and all patients at timepoint of post consolidation 3 had negative MRD (Fig. 8C). No significant differences were observed among the four different course of treatment in assay sensitivity (Fig. 8D).

We also compared the PML-RARa/ABL NCN, MRD value and assay sensitivity of 3-plex RT-qPCR assay with single RT-qPCR assay in 199 BM samples of patients with APL after induction and 3 cycles of consolidation. No significant differences between 3-plex RT-qPCR assay and single RT-qPCR assay were observed in the PML-RARa/ABL NCN, MRD values and assay sensitivity for patients with APL after induction and consolidation (*P* > 0.05) (S2A-S2C Fig.). The 3-plex RT-qPCR assay was highly correlated with the single RT-qPCR assay in the MRD values for patient with APL during follow-up (Spearman’s rho coefficient = 0.998, *P* < 0.01) (S2D Fig.). Results indicate that, compared with Single RT-qPCR, the 3-plex RT-qPCR has similar performance in MRD monitoring for patients with APL.

## Discussion

Individuals with APL usually express 1 of 3 PML-RARa transcripts associated with t(15;17). APL patients can benefit from serial MRD monitoring as they are treated pre-emptively at the point of molecular relapse in comparison to frank relapse [15,29]. We developed a quantitative 3-plex RT-qPCR assay to detect and monitor the three PML-RARa transcripts, regardless of isoforms, for APL patients. The primers and probe were designed using a common forward primer targeting to the critical breakpoint of PML exon 6 (breakpoint at 1579). The optimized 3-plex RT-qPCR showed a relatively high specificity, sensitivity, and reproducibility, which can be used for fast diagnosis and MRD monitoring of APL with PML-RARa without false-negative results for some rare variants of PML-RARa bcr2. Moreover, the 3-plex RT-qPCR assay is convenient and cost-effective for potential use in hospital laboratories.

Some studies reported that HybProbe-based RT-qPCR assays allow the detection of the PML-RARa fusion transcripts [30,31]. TaqMan-based RT-qPCR is the most appropriate method in clinical diagnostic laboratories considering cost, turnaround time, and assay performance [32]. To date, the RT-qPCR methods based on the TaqMan system using multiple primers have been employed to quantitatively detect three PML-RARa transcripts [17,18]. These RT-qPCR assays are still laborious and costly because multiple primers, probes, and reactions are needed in identifying each of the three transcripts. In this study, the 3-plex RT-qPCR assay is a cost-saving and convenient method for the simultaneous detection and quantification of the three PML-RARa transcripts in a single reaction, achieving fast diagnosis of APL with PML-RARa. The use of TaqMan MGB probe and subsequent optimization provided a flexible system and enabled the incorporation of three primers and one probe to simultaneously detect all three PML-RARa transcripts.

Accurate standard curves are essential for the precise quantification of gene expression when employing RT-qPCR method. In this study, the standard curves showed a very high correlation coefficient (more than 0.99) and provided reliable quantification within the diagnostic range (from 10^6^ to 10 copies/reaction). Therefore, one set of plasmid standards (bcr1) was prepared as calibrators to establish NCN for the transcript levels because the efficiencies estimated from the slopes of the calibration curves for the PML-RARa bcr1, bcr2, and bcr3 were very similar. Furthermore, the serial diluted clinical samples with high concentration of PML-RARa displayed similar kinetics and optimal PCR amplification (data not shown). In any case, we emphasized the strict manipulation rules according to the Minimum Information for Publication of Quantitative Real-Time PCR Experiments (MIQE) guidelines [33]. The developed assay showed a sensitivity of 10 copies plasmid or 10^-4^ RNA cell line dilution for PML-RARa, and this sensitivity is comparable to other studies [17,18,31]. Moreover the CVs of inter- and intra-assays were less than 3% for all the three transcripts, indicating that this assay is sensitive and reliable for PML-RARa detection. The results generated using the 3-plex RT-qPCR assay completely agreed with those of the traditional methods used for the detection of the three PML-RARa transcripts associated with t(15;17) in a series of 319 patient samples, suggesting that the assay is specific for the detection of PML-RARa fusion gene. In the 61 diagnosed APL samples, 3-plex RT-qPCR revealed that no significant differences were observed in the Ct values and NCN of the PML-RARa among the different types of transcripts (Fig. 5C and 5D).

A previous research identified nucleotide 1685 as the most common breakpoint found in PML-RARa bcr2 positive samples [34]. The 3-plex RT-qPCR and single RT-qPCR exhibited high correlation and similar assay sensitivity for most of the PML-RARa positive samples. However, the 3-plex RT-qPCR detected one case of PML-RARa bcr2 (with breakpoint at 1579 on PML exon 6) that was not detected using the single RT-qPCR. Some atypical bcr2 transcript with high copies of PML-RARa could not be detected with EAC bcr1 and bcr2 primers but could be suspected for a late amplification with bcr3 primers. Nonetheless, when EAC method was applied to detect the atypical bcr2 transcript with relatively low copies of PML-RARa, the amplification curve still was under the threshold, which was confirmed on the case of PML-RARa bcr2 verified by sequencing in this study. Although single RT-qPCR is the standardized protocol for PML-RARa transcripts, it was designed for MRD studies and the EAC primer and probe sets have not been designed for the detection of PML-RARa variant at initial diagnosis [18]. The single RT-qPCR for PML-RARa, established in 2003, does not detect the rare transcripts of PML-RARa. Consequently, the EAC sets could lead to false-negative results and/or less efficiency of PCR amplifications, and the latter may be mistaken for a nonspecific amplification, resulting in a third of the false negative result for the detection of PML-RARa bcr2 (33.3%) in the present study by the single RT-qPCR when used for screening APL during diagnosis. Therefore, the 3-plex RT-qPCR may be more efficient than single RT-qPCR in identifying the APL cases with PML-RARa bcr2 at initial diagnosis. We can speculate that the integration 3-plex RT-qPCR in APL diagnosis may result in the detection of uncommon breakpoints in the future. False negative results may also be avoided.

MRD monitoring is becoming increasingly significant within clinical trials to measure response to ATRA-based therapies and to predict or identify relapse of APL that prompts treatment modification. In the present study, results of MRD monitoring in 57 patients with APL after induction and 3 cycles of consolidation therapy were very similar to previous findings reported by David Grimwade et al. [15], implying that the 3-plex RT-qPCR assay can be used for MRD monitoring. In despite of a slightly different results in in the 3rd cycle of consolidation (4% positivity of PML-RARa), this difference may be due to our relatively small sample size and population selection. Furthermore, compared with Single RT-qPCR, the 3-plex RT-qPCR has similar performance in MRD monitoring for patients with APL.

RT-qPCR can be performed as either a two-step or one-step procedure with their corresponding advantages. A one-step, multiplex, real-time RT-PCR with four probes and three primers has been described for detection of PML-RARa [17], which uses the beta-actin control gene to normalize the results. This assay may minimize turnaround time in the reverse transcription process, but it is expensive and beta-actin acts as a competitive control amplified with PML-RARa bcr3, resulting in the possible decline in amplification efficiency. In the 3-plex RT-qPCR assay, we used a two-step procedure to allow both the reverse transcription and real-time PCR to be performed under optimal conditions. A two-step procedure exhibited a better assay sensitivity than a one-step procedure (data not shown). ABL may be a more acceptable endogenous control gene than beta-actin for molecular diagnosis of leukemia [27]. The use of ABL control enabled results to be normalized according to the amount of RNA and RT efficacy. Experimental sensitivity per sample and MRD value can also be calculated according to the results of PML-RARa and ABL [18,27], which is particularly important for PCR negative follow-up samples.

Studies showed that clinical relevance of APL may be associated with the specific type of transcript expressed in an individual. A limitation of our assay is that the quantification of PML-RARa transcript is not isoform-specific. However, specific types of PML-RARa fusion transcripts can be verified by complementary use of gel electrophoresis of the PCR products (S1 Fig.). The major advantage of this system is that it permits the simultaneous detection of the three PML-RARa transcripts associated with APL in a relatively short time, which is critical to patient care. Although RT-qPCR has been used for MRD assessment, conventional nested RT-PCR is still being used in screening most fusion transcripts at present. Once the suspected samples are confirmed for PML-RARa positive by conventional nested RT-PCR, quantitative data of PML-RARa transcripts can be obtained by our assay in 75 min using the same cDNA samples. Though the results of our assay showed 100% sensitivity and 100% specificity in 319 BM samples of patients with leukemia, more samples from multi centers would be needed to verify our results. Given the design of universal primers and probe, this technique could certainly be applied to the detection of other fusion genes with multiple transcripts, such as BCR-ABL and CBFB-MYH11.

In conclusion, this novel 3-plex RT-qPCR assay is a sensitive and specific assay for identifying APL with PML-RARa and monitoring MRD. Its applicability in molecular diagnostic laboratories is supported by the rapidness, simplicity and efficiency of the procedure, which effectively complements cytogenetic, FISH analyses and conventional nested RT-PCR, and may yield significant financial savings. This is a critical assay to assist in the urgent diagnosis of suspected APL and in reaching an objective decision on whether to initiate the treatment with ATRA. Our 3-plex RT-qPCR can be also used to quantify changes in PML-RARa expression during treatment and to predict the possibility of relapse.

## Supporting Information

S1 Fig Verification of three PML-RARa transcript subtypes by complementary use of gel electrophoresis of amplification products after 3-plex RT-qPCR. Amplification products of the selected samples were electrophoresed on 2% agarose gel and stained in the 0.5% nucleic acid dyes Goldview. White arrows indicate the target transcripts of PML-RARa bcr1, bcr2 and bcr3 (bcr1: 288 bp; bcr2: 236bp; bcr3: 113 bp); Black arrows indicate alternative spliced bands. Lane 1 and 2 indicate PML-RARa bcr1; Lane 3 and 4 indicate PML-RARa bcr 3; Lane 5 indicates PML-RARa bcr2; Lane 6 indicates no-template controls (NTC); M indicates 100bp size marker.
(TIF)

S2 Fig Comparison of 3-plex RT-qPCR assay and single RT-qPCR assay for MRD monitoring. (A) Comparison of PML-RARa/ABL NCN of patients with APL after induction and consolidation between 3-plex RT-qPCR assay and single RT-qPCR assay. (B) Assay sensitivity of APL samples after induction and consolidation by 3-plex RT-qPCR assay and single RT-qPCR assay. (C) MRD assessment of patients with APL after induction and consolidation by 3-plex RT-qPCR assay and single RT-qPCR assay. (D) Correlation of MRD values of patients with APL after induction and consolidation by 3-plex RT-qPCR assay and single RT-qPCR assay. There were no significant differences in the PML-RARa/ABL NCN, MRD values and assay sensitivity for patients with APL during follow-up between 3-plex RT-qPCR assay and single RT-qPCR assay (*P* > 0.05).
(TIF)
