# Supplementary figures and images for: Development and Validation of a 3-Plex RT-qPCR Assay for the Simultaneous Detection and Quantitation of the Three PML-RARa Fusion Transcripts in Acute Promyelocytic Leukemia
Source: PLoS One. 2015 Mar 27;10(3):e0122530. doi: 10.1371/journal.pone.0122530 (PMC4376893; doi:10.1371/journal.pone.0122530)

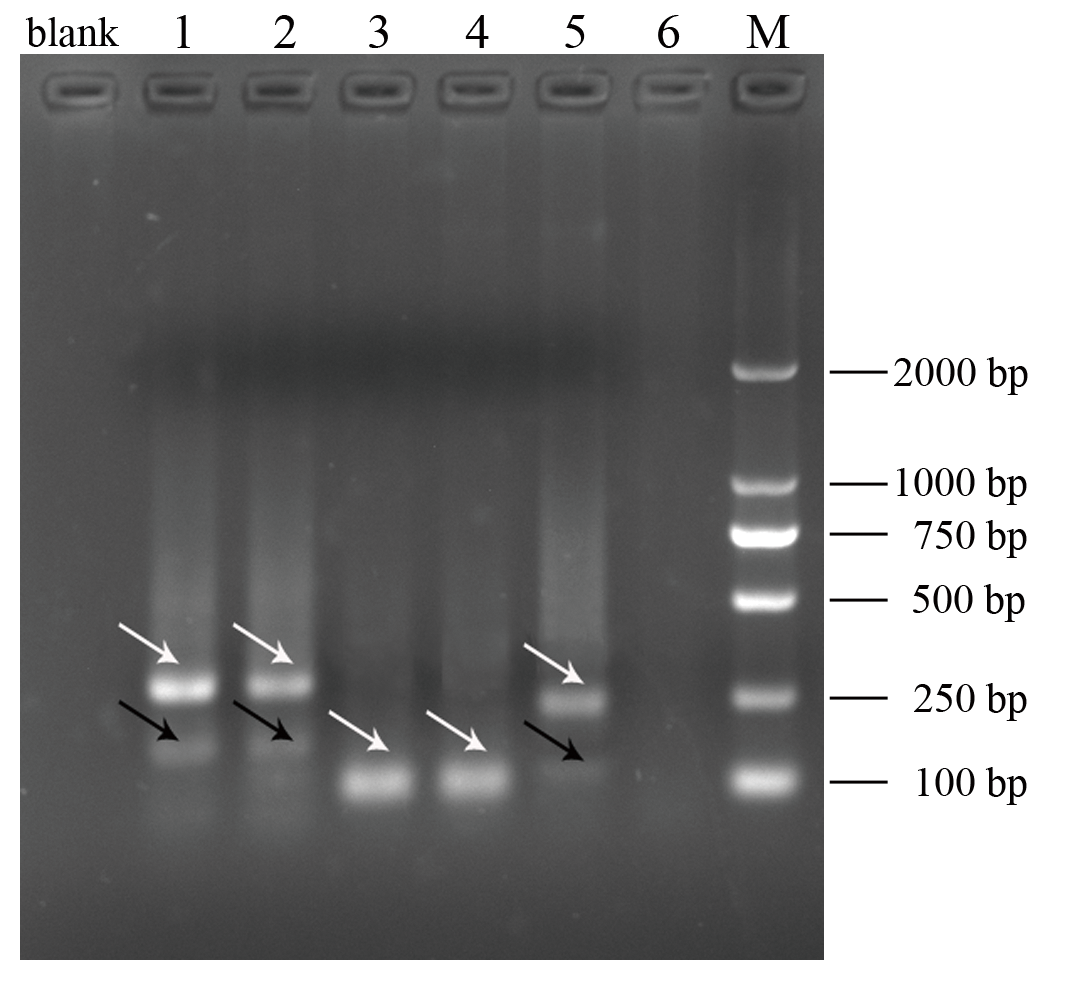

Supplement: S1 Fig — Amplification products of the selected samples were electrophoresed on 2% agarose gel and stained in the 0.5% nucleic acid dyes Goldview. White arrows indicate the target transcripts of PML-RARa bcr1, bcr2 and bcr3 (bcr1: 288 bp; bcr2: 236bp; bcr3: 113 bp); Black arrows indicate alternative spliced bands. Lane 1 and 2 indicate PML-RARa bcr1; Lane 3 and 4 indicate PML-RARa bcr 3; Lane 5 indicates PML-RARa bcr2; Lane 6 indicates no-template controls (NTC); M indicates 100bp size marker. (TIF) [file pone.0122530.s001.tif]

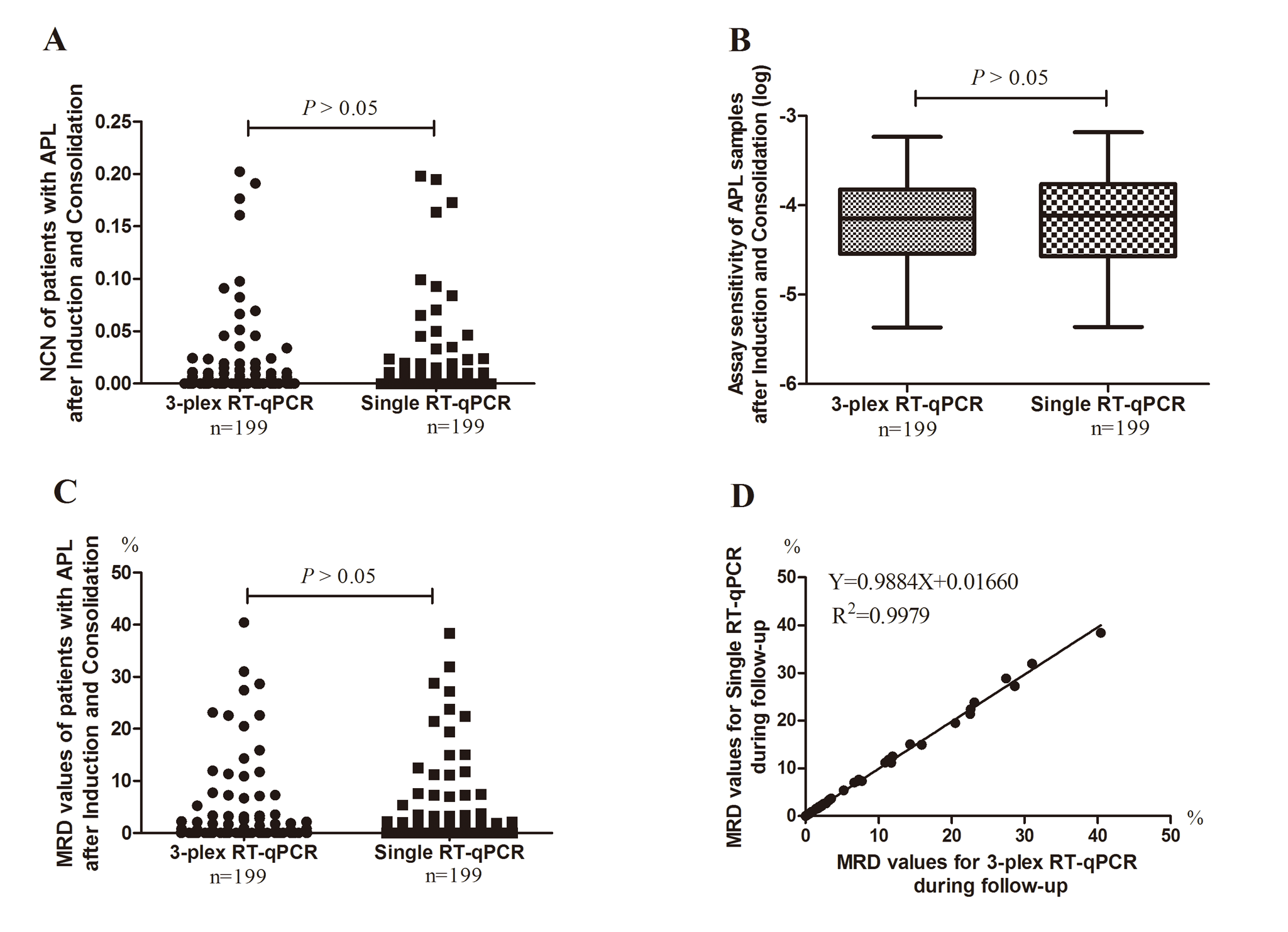

Supplement: S2 Fig — (A) Comparison of PML-RARa/ABL NCN of patients with APL after induction and consolidation between 3-plex RT-qPCR assay and single RT-qPCR assay. (B) Assay sensitivity of APL samples after induction and consolidation by 3-plex RT-qPCR assay and single RT-qPCR assay. (C) MRD assessment of patients with APL after induction and consolidation by 3-plex RT-qPCR assay and single RT-qPCR assay. (D) Correlation of MRD values of patients with APL after induction and consolidation by 3-plex RT-qPCR assay and single RT-qPCR assay. There were no significant differences in the PML-RARa/ABL NCN, MRD values and assay sensitivity for patients with APL during follow-up between 3-plex RT-qPCR assay and single RT-qPCR assay (P > 0.05). (TIF) [file pone.0122530.s002.tif]
